# Role of Matrix Metalloproteinases in Myelin Abnormalities and Mechanical Allodynia in Rodents with Diabetic Neuropathy

**DOI:** 10.14336/AD.2021.0126

**Published:** 2021-10-01

**Authors:** Xueting Deng, Pingchuan Ma, Mingzheng Wu, Huabao Liao, Xue-Jun Song

**Affiliations:** ^1^SUSTech Center for Pain Medicine, School of Medicine, Southern University of Science and Technology, Shenzhen, China.; ^2^Medical Center for Digestive Diseases, Second Affiliated Hospital, Nanjing Medical University, Nanjing, Jiangsu, China.; ^3^Department of Perioperative Medicine, SUSTech Hospital, Southern University of Science and Technology, Shenzhen, China.

**Keywords:** diabetic neuropathic pain, MMP-9, MMP-2, α-Lipoic acid, Myelin abnormalities

## Abstract

The treatment of diabetic neuropathic pain (DNP) is a major clinical challenge. The underlying mechanisms of diabetic neuropathy remain unclear, and treatment approaches are limited. Here, we report that the gelatinases MMP-9 and MMP-2 play a critical role in axonal demyelination and DNP in rodents. MMP-9 may contribute to streptozotocin (STZ)-induced DNP via inducing axonal demyelination and spinal central sensitization, while MMP-2 may serve as a negative regulator. In STZ-induced DNP rats, the activity of MMP-9 was increased, while MMP-2 was decreased in the dorsal root ganglion and spinal cord. Spinal inhibition of MMP-9, but not MMP-2, greatly suppressed the behavioral and neurochemical signs of DNP, while administration of MMP-2 alleviated mechanical allodynia. In mice, STZ treatment resulted in axonal demyelination in the peripheral sciatic nerves and spinal dorsal horn, in addition to mechanical allodynia. These neuropathic alterations were significantly reduced in MMP-9^-/-^ mice. Finally, systematic administration of α-lipoic acid significantly suppressed STZ-induced mechanical allodynia by inhibiting MMP-9 and rescuing MMP-2 activity. These findings support a new mechanism underlying the pathogenesis of diabetic neuropathy and suggest a potential target for DNP treatment. Gelatinases MMP-9 and MMP-2 play a critical role in the pathogenesis of diabetic neuropathy and may serve as a potential treatment target. MMP-9/2 underlies the mechanism of α-lipoic acid in diabetic neuropathy, providing a potential target for the development of novel analgesic and anti-inflammatory drugs.

Diabetic neuropathic pain (DNP) is an intractable clinical complication of diabetes mellitus that affects millions of patients with diabetes [1-3]. DNP is characterized by spontaneous painful sensations (*e.g*., burning or sharp pain) and cutaneous allodynia, severely impacting patients’ quality of life and causing mood disturbances [3, 4]. However, the specific cellular and molecular mechanisms underlying the pathogenesis of DNP remain unknown. Previous studies have implicated that hyperglycemia-induced nerve damage and the production of proinflammatory cytokines play a pivotal role in the pathophysiology of DNP [5-8]. Notably, myelin abnormalities have been consistently observed in patients with diabetes and animal models of DNP [9, 10]. Damaged myelination in afferent nerve fibers may induce dysfunction in nociceptive transduction, resulting in hyperalgesia and allodynia in DNP [11-15]. Other mechanisms that contribute to the development of DNP involve diabetes-related oxidative stress and immune dysfunction [1, 5, 15].

Matrix metalloproteinases (MMPs), which belong to the metzincin clan of the metalloproteinase superfamily, are widely implicated in inflammation and tissue remodeling. In the nervous system, MMPs are expressed in both neurons and glial cells, regulating their functions through the cleavage of extracellular matrix proteins, cytokines, and chemokines. Emerging evidence has shown that MMPs regulate neuroinflammatory processes in response to injuries in the peripheral and central nervous systems [16-20]. In particular, gelatinases MMP-9 and MMP-2 play distinct roles in the development of neuropathic pain after peripheral nerve injury [17]. In addition, we demonstrated that spinal MMP-9 contributes to morphine withdrawal symptoms and morphine tolerance [21]. In experimental diabetic neuropathy, the involvement of MMP-2 and MMP-9 has also been implicated [22-26]. However, the role that MMPs play in DNP-associated mechanical allodynia and myelin abnormalities remains unknown.

In this study, we hypothesized and have provided evidence that MMP-9 and MMP-2 in the dorsal root ganglion (DRG) and dorsal horn (DH) of the spinal cord may play an important role in the pathogenesis of diabetic neuropathy and associated DNP. This finding also suggests a potential target for DNP treatment. Furthermore, we have confirmed that the natural antioxidant (±)-α-lipoic acid (α-LA), which has been exploited for decades to treat diabetic peripheral neuropathy, can greatly suppress DNP by regulating MMP-9 and MMP-2 activity. This finding provides mechanistic insights into the action of a therapeutically promising compound derived from natural products.

## MATERIALS AND METHODS

### Animals, drugs and drug administration

Adult male Sprague-Dawley rats (200-220 g-wt) were purchased from SUSTech Animal Center, FVB mice (20-22 g-wt) from Nanjing University Model Animal Research Center, and MMP-9^-/-^ mice (20-22 g-wt) from Jackson Laboratories. All animals were used in accordance with the regulations of the Ethics Committee of the International Association for the Study of Pain, and all protocols (SUSTC-JY2017033) were approved by the Institutional Animal Care and Use Committees of the Southern University of Science and Technology. MMP-9 inhibitor (MMP-9i), MMP-2 inhibitor (MMP-2i), and MMP-2 were purchased from Calbiochem (Millipore). MMP-9i and MMP-2i, each in 10 μg, were dissolved in phosphate buffer solution (PBS) and then injected intrathecally (i.t., 20 μL) by lumbar puncture at the intervertebral space of L4-5 and L5-6 for multiple injections in rats. MMP-2 (0.2, 0.4, and 0.8 pmol, i.t.) was administered to wild type (WT) and MMP-9^-/-^ mice, respectively. The α-LA was purchased from Sigma and was first dissolved in saline and then injected intraperitoneally (i.p., 0.1 ml/10 g-wt).

### Animal model of diabetes in rats and mice

Diabetes was induced by a single streptozotocin (STZ)(Sigma) of 70 mg/kg i.p. in rats [27, 28] and 200 mg/kg in mice [29, 30], freshly dissolved in 0.1 mol/L citric acid buffer (CAB, pH 4.5). Animals in the control group were injected with an equivalent volume of citrate buffer. Single or multiple measurements of blood glucose were made after STZ administration in each animal. Blood samples were collected from the tail vein blood vessels. The onset of diabetic conditions was defined as glucose levels >16.6 mmol/L.

### Assessment of mechanical allodynia

Mechanical allodynia was indicated by a significant decrease in the threshold of paw withdrawal in response to mechanical indentation of the plantar surface of each hind paw with a sharp, cylindrical probe with a uniform tip diameter of 0.2 mm provided by an Electrovonfrey (Almemo 2390-5, Anesthesiometer IITC). The probe was applied to six designated loci distributed over the plantar surface of the hind paw. The minimal force (in mN) that induced paw withdrawal was recorded on the display. The threshold of mechanical withdrawal in each animal was calculated by averaging the four readings, and the force was converted into millinewtons.

### Gelatin zymography

The animals were deeply anesthetized and transcardially perfused with PBS, and then the segments of L4-L5 spinal DH and L4-L5 DRG were rapidly dissected and homogenized in a lysis buffer. Aliquots (10 μL) of the homogenates were saved for total protein measurements. The homogenates were centrifuged and incubated with gelatin-Sepharose 4 B (Pharmacia Biotech). After incubation, the pellets were resuspended in an elution buffer. The entire sample was loaded onto a sodium dodecyl sulfate gel containing gelatin. The gel was washed and incubated in an incubation buffer. Finally, zones of gelatin degradation representing proteolytic activity were identified by staining the gel with Coomassie Blue. This protocol was similar to that previously described [21].

**Figure 1.**
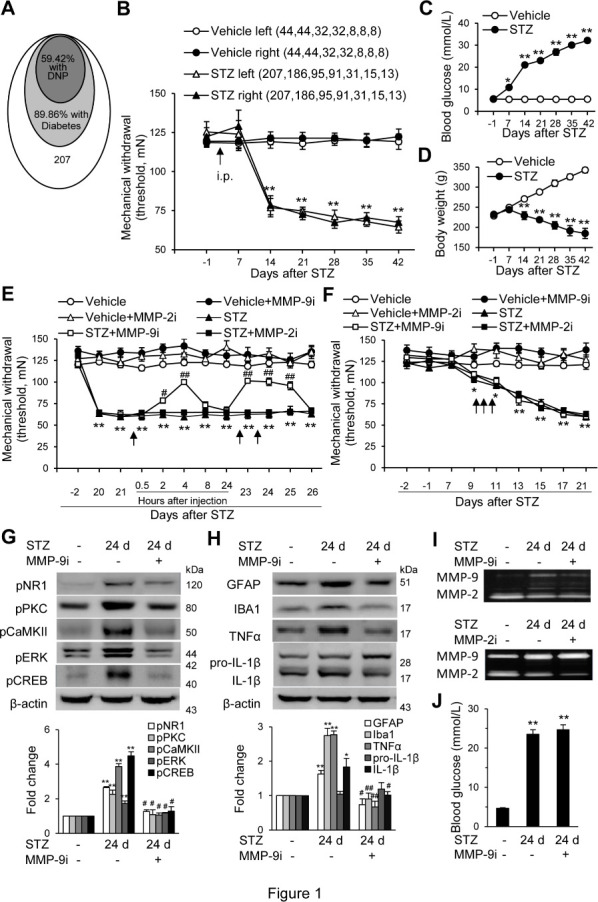
The spinal administration of the MMP-9 inhibitor MMP-9i, but not the MMP-2 inhibitor MMP-2i, suppresses behavioral and neurochemical signs of DNP induced by i.p. STZ (70 mg/kg) in rats. (A) The proportion of animals that developed diabetes was defined as those with high blood glucose (>16.6 mmol/L) and DNP with mechanical allodynia. The animals without high blood glucose did not show DNP. (B) The time course of the changes in the mechanical threshold of the hind paw following STZ treatment. The number of animals corresponding to each of the testing points in each group is indicated in parentheses. (C, D) The time course of the blood glucose changes (C) and body weight (D) following STZ treatment. All the rats included in C and D exhibited mechanical allodynia. (E) A single dose of MMP-9i, but not MMP-2i, each in 10 μg/20 μl at a later phase, produced immediate- and accumulated-inhibition of mechanical allodynia. (F) Repeated MMP-9i or MMP-2i (10 μg in 20 μl, daily for three consecutive days in the early phase) did not prevent or inhibit the development of mechanical allodynia. Eight rats were included in each group, and all the STZ-treated animals were first identified with high blood glucose (>16 mmol/L). (G, H) Spinal administration of MMP-9i suppresses the hyperactivity of neurochemical signals in the DNP and DH. Western blot analysis showing phosphorylation of NR1, PKC, CaMKII, ERK, and CREB (G) as well as activation of the glial cells (GFAP and Iba1), IL-1β, and TNF-α (H) and inhibitory effects of MMP-9i on these alterations. Four to five samples were included in each of the groups. (I) Gelatin zymography showing the changes of activity of MMP-9 and MMP-2 in the spinal cord following MMP-9i and MMP-2i treatment, respectively. (J) Blood glucose levels measured after the injection of MMP-9i 24 days after the injection of STZ. Data are expressed as the mean ± SEM. Two-way (B-F) or one-way ANOVA (G-H, J), *p < 0.05, **P < 0.01 versus vehicle; #P< 0.05, ##P < 0.01 versus the testing point immediately before the drug administration (E, F, J); #p < 0.05, ##p < 0.01 versus STZ (G, H).

### Western blotting and immunohistochemistry

Immunoblotting was performed with antibodies against pNR1 (1:1000, Cell Signaling), pPKC (1:1000, Cell Signaling), pCaMKII (1:800, Cell Signaling), pERK (1:1500, Cell Signaling), pCREB (1:1000, Cell Signaling), GFAP (1:2000, Millipore), IBA1 (1: 1000, Abcam), IL-1β (1:1000, Santa Cruz Technology), TNF-α (1:1000, Cell Signaling), and β-actin (1:2000, Bioworld). Immunohistochemistry was performed as previously described [21, 31-33].

### Electron microscopy

Small blocks from the mouse sciatic nerve and L4-L5 segments of the DH were processed for electron microscopy and embedded in Epon resin. The ultrathin tissue sections obtained were contrasted with uranyl acetate and lead citrate and examined under a transmission electron microscope (JEM-1010, JEOL). The ultrastructural features of the myelin sheaths were semi-quantitatively analyzed after the observation of a minimum of 100 randomly selected myelinated axons per group in three different grids (100 axons per grid). Then, a percentage was established for each of them.

### Statistical analysis

SPSS version 15 was used to conduct all the statistical analyses. A one-way and two-way analysis of variance (ANOVA) with repeated measures followed by Bonferroni post hoc tests were used to examine the alteration of the expression of the detected proteins between groups and the behavioral responses to mechanical stimuli over time, respectively. Results are expressed as the mean ± SEM. Statistical significance was set at a p-value of < 0.05.

## RESULTS

### High blood glucose and mechanical hypersensitivity induced by i.p. STZ

We started by establishing a rat model of DNP using i.p. STZ. Following STZ treatment (70 mg/kg, n=207), 89.86% of animals developed diabetes with high blood glucose (>16.6 mmol/L), and 59.42% developed DNP manifested as mechanical allodynia. Animals without diabetes did not exhibit DNP. The data are summarized in Fig. 1A. DNP was first detected on day 14 after STZ treatment and was maintained for seven weeks, the last time point tested (Fig. 1B). Significantly increased blood glucose was observed seven days after the STZ injection. The average blood glucose level increased to 10-20 mmol/L in the second week and continued to increase in a linear manner in the following two to six weeks (Fig. 1C). All animals with high glucose and mechanical allodynia showed gradually decreasing body weight during the two-to-six-week period (Fig. 1D). The time courses of the changes in mechanical allodynia, blood glucose, and body weight loss were well matched following STZ treatment (Fig. 1B-D). Meanwhile, the STZ-treated rats or mice with mechanical allodynia did not show significant thermal hypersensitivity (data not shown). This was consistent with previous reports that STZ-diabetic rats or mice did not develop significant thermal hypersensitivity or hyperalgesia [34, 35]. Among the animals that received i.p. STZ, only those that exhibited both high blood glucose and mechanical allodynia were included in the following analyses. These results demonstrated that approximately 60% of rats that received i.p. STZ developed DNP, confirming the findings of our recent report [28]. Again, this emphasizes the limited success rate of STZ-induced DNP, suggesting a valuable reference for performing similar research using this model.

### Spinal inhibition of MMP-9, but not MMP-2, suppresses the behavioral and neurochemical signs of DNP

To test our hypothesis that MMP-9 and MMP-2 might be involved in DNP, we first examined the effects of the spinal inhibition of MMP-9 and MMP-2 activity on DNP and the neurochemical alterations associated with DNP. We found that a single dose of MMP-9 inhibitor MMP-9i (10 μg/20 μL, i.t.) significantly inhibited the established ongoing mechanical allodynia. The inhibition started within 2 h, peaked at 4 h, and returned to baseline within 8 h. Following multiple treatments of MMP-9i, administered at the same dose at 24 h and 48 h after the first injection, the results showed an accumulated inhibitory effect on mechanical allodynia, and the inhibition lasted for at least 36 h after the third administration (Fig. 1E). In contrast, the MMP-2 inhibitor MMP-2i (10 μg/20 μL) did not produce any effects on mechanical allodynia (Fig. 1E). To further test whether MMP-9 or MMP-2 inhibitors would affect the induction/production of DNP, the animals were treated with MMP-9i or MMP-2i in the early phase after STZ injection when DNP was not yet well developed. The results showed that daily i.t. MMP-9i or MMP-2i, each in 10 μg/20 μL, for three consecutive days after STZ treatment (days eight to ten), failed to prevent the development of DNP (Fig. 1F). These results demonstrate that MMP-9, and not MMP-2, in the spinal cord and DRG, may contribute to maintaining the established DNP, while MMP-9 and MMP-2 are not important for the induction of DNP.

N-methyl-D-aspartate receptor (NMDAR) activation and the subsequent activation of Ca^2+^-dependent downstream signaling pathways [36, 37], as well as the activation of microglia and astrocytes and proinflammatory cytokines, play critical roles in neuropathic pain [36-38]. The western blotting analysis showed that in rats with DNP, the expression of phosphorylation of NR1, ERK, PKC, CaMKII, and CREB was significantly increased in the DH. Repetitive treatment with MMP-9i (10 μg/20 μL, i.t., daily for three subsequent days), 21, 22, and 23 days after STZ treatment when both the expression of MMP-9 and mechanical allodynia had been well developed significantly inhibited the increased phosphorylation of these molecules (Fig. 1G). Meanwhile, microglial cells (IBA1) and astrocytes (GFAP), as well as the proinflammatory cytokines TNF-α and IL-1β in the DH, were activated (Fig. 1H). Repetitive spinal administration of MMP-9i (with the same protocols as the treatment described above) significantly inhibited the increased activation of IBA1, GFAP, TNF-α, and IL-1β (Fig. 1G, H). Meanwhile, MMP-9i and MMP-2i also inhibited the activity of the corresponding gelatinases MMP-9 and MMP-2 in the spinal cord following STZ treatment (Fig. 1I). STZ-induced high blood glucose levels were not changed by MMP-9i treatment (Fig. 1J). These results support that MMP-9, but not MMP-2 plays an important role in DNP.

**Figure 2.**
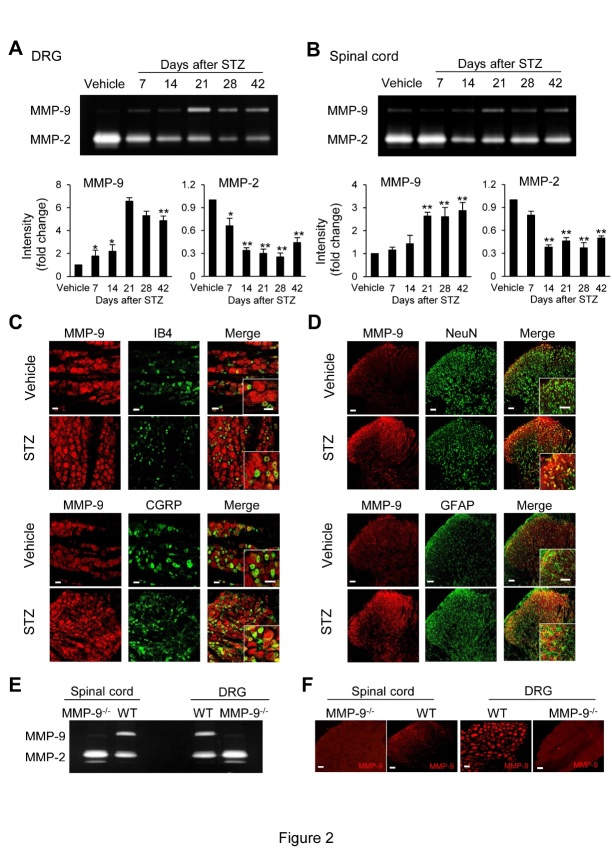
STZ treatment increases MMP-9 activity and decreases MMP-2 activity in the DRG and DH. (A, B) Gelatin zymography showing the time course of the changes of activity of MMP-9 and MMP-2 in the DRG (A) and DH (B). Five to six samples were included in each of the groups in the gelatin zymography analysis. *p < 0.05, **p < 0.01 vs vehicle control. (C) Confocal images of immunostaining showing the expression of MMP-9 in the DRG and its colocalization with IB4- and CGRP-positive DRG cells. (D) Confocal image of immunostaining showing the expression of MMP-9 and its colocalization with neurons (NeuN) and astrocytes (GFAP) in the DH. (E, F) The specificity of MMP-9 was confirmed in the DRG and spinal cord from MMP-9^-/-^ mice by gelatin zymography (E) and confocal images of immunostaining (F), respectively. Original magnification: × 200, × 800 (merge); scale bar = 50 µm in C, D, and F.

**Figure 3.**
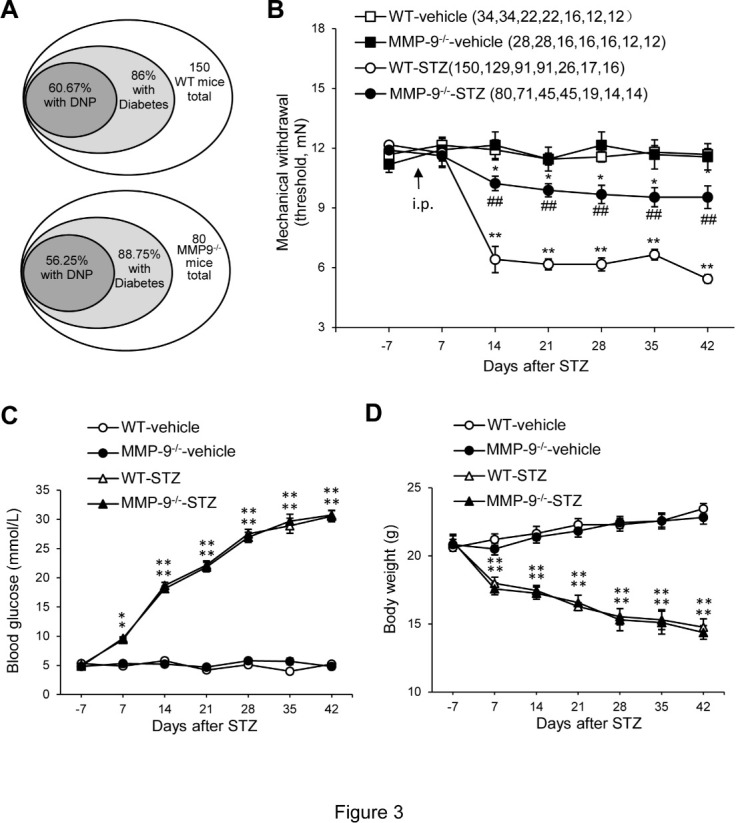
Mechanical hypersensitivity allodynia, high blood glucose, and body weight loss following i.p. STZ (200 mg/kg) in WT and MMP-9^-/-^ mice. (A) The proportion of animals that developed diabetes was defined as those with high blood glucose (>16.6 mmol/L) and DNP with mechanical allodynia. The animals without high blood glucose did not show DNP. (B) The time course of the changes of the mechanical threshold of the hind paw following STZ treatment. The number of animals corresponding to each of the testing points in each group is indicated in parentheses. (C) The time course of the changes in blood glucose following STZ treatment. (D) Time course of the changes in body weight following STZ treatment. All the mice included in C and D exhibited mechanical allodynia. Data are expressed as the mean ± SEM. Two-way ANOVA, *p < 0.05, **p < 0.01 versus Vehicle control (B-D).

### Activity of MMP-9 and MMP-2 in the DRG and the DH after STZ treatment

We examined the activity of MMP-9 and MMP-2 in the DRG and DH in rats with DNP using gelatin zymography. We found that following STZ treatment, the activity of MMP-9 was greatly increased, while MMP-2 was decreased in the DRG and DH. In the DRG, MMP-9 activity was moderately increased in the first two weeks, followed by a sudden increase from week three (Fig. 2A). In the DH, MMP-9 activity was unchanged in the first two weeks but experienced a sudden increase (4-6-fold) during weeks three to six (Fig. 2B). We then examined the distributions of MMP-9 in the DRG and DH. MMP-2 was not examined since it was significantly decreased. MMP-9 protein immunoreactivity was found in both the nonpeptidergic isolectin B4 (IB4) and the peptidergic calcitonin gene-related peptide (CGRP)-positive nociceptive small DRG neurons (Fig. 2C). In the DH, MMP-9 immunoreactivity was colocalized primarily with neuronal somata (NeuN) and astrocytes (GFAP) (Fig. 2D). The specificity of the MMP-9 antibody in the DRG and spinal cord tissues was also confirmed in MMP-9^-/-^ mice (Fig. 2E, F).

**Figure 4.**
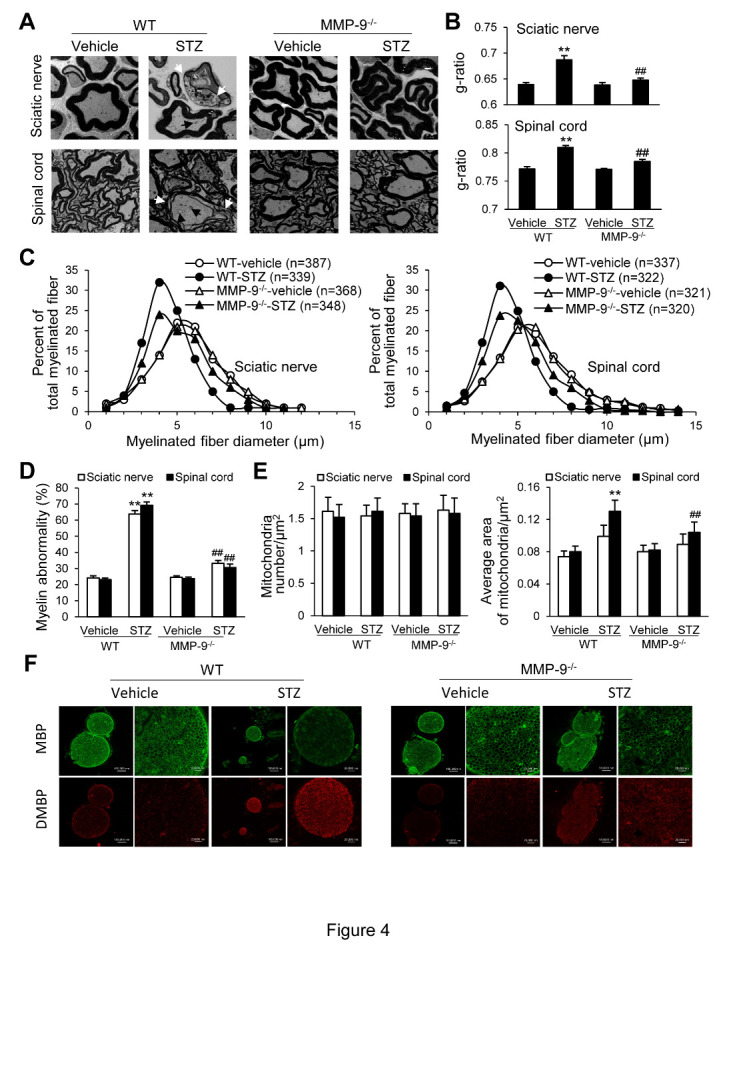
Electron micrographs showing signs of axonal degeneration and myelin abnormalities in the sciatic nerve and DH in WT and MMP-9^-/-^ mice after STZ treatment. (A) Representative electron micrographs. Scale bar = 2 µm. (B) The g-ratio was determined by dividing the myelinated axon diameter by the fiber diameter (n = 6) [55]. (C) Distributions of the myelinated fibers based on their axonal sizes. (D) Myelin abnormalities including irregular fiber shapes, myelin infoldings (myelin invaginations in the axoplasm), and outfoldings (myelin evaginations in the Schwann cell cytoplasm). (E) Average numbers and area of mitochondria. In B-E, each group contained three or four sciatic nerves or the DH samples from independent animals, and a total of 300-400 fibers were observed from these nerve tissues, taken 24 days after the STZ treatment. Data in B-E are expressed as the mean ± SEM. One-way ANOVA, **p < 0.01 versus vehicle; ##p < 0.01 versus STZ in WT. (F) Confocal images of immunostaining showing the expression of myelin basic protein (MBP) and degraded MBP (DMBP) in the sciatic nerve following STZ treatment in WT and MMP-9^-/-^ mice.

### Targeted mutation of MMP-9 attenuates DNP

Given the pharmacological evidence that blocking MMP-9 in the spinal cord can alleviate DNP in rats, we further investigated the effects of MMP-9 mutation on DNP in mice. Following STZ treatment (200 mg/kg, i.p.), 88.75% (n=80) of the MMP-9^-/-^ mice developed hyperglycemia (vs. 86%, n=150 wild-type, WT) (Fig. 3A) and 56.25% (n=80) of the MMP-9^-/-^ mice developed mechanical allodynia (vs. 60.67%, n=150 WT) (Fig. 3A). However, in approximately 52%-100% of MMP-9^-/-^ mice (n=80), the severity of DNP-related mechanical allodynia was significantly alleviated (Fig. 3B). Similar levels of high blood glucose (Fig. 3C) and body weight loss (Fig. 3D) were observed in MMP-9^-/-^ and WT mice following STZ treatment. These results indicate that targeted mutation of MMP-9 can greatly suppress STZ-induced DNP, confirming the pharmacological inhibitory effects of spinal blocking MMP-9.

**Figure 5.**
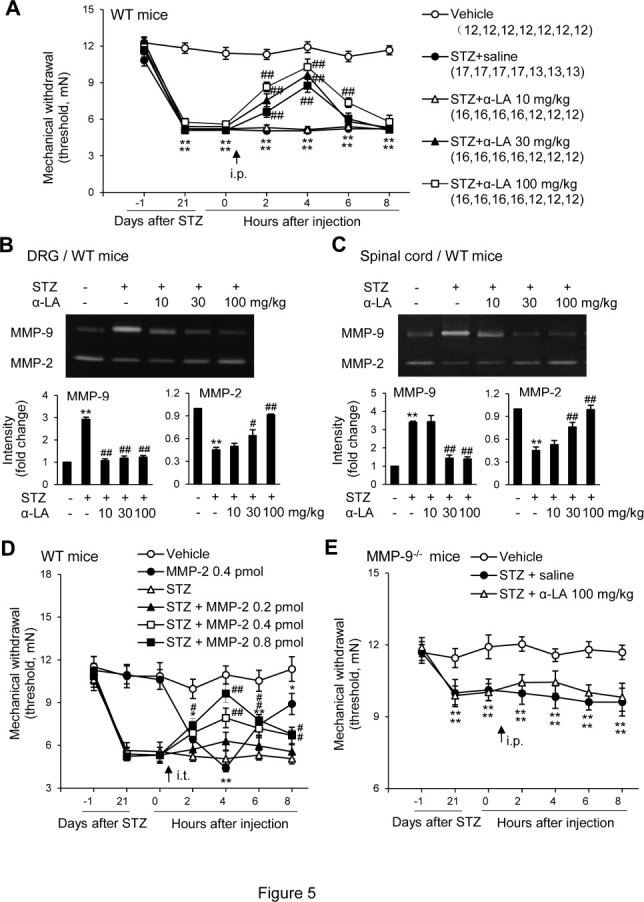
The systematic administration of α-LA suppresses STZ-induced mechanical allodynia, inhibits the activity of MMP-9, and rescued the deficit of MMP-2 activity in the spinal cord and DRG in WT mice. (A) A single injection of α-LA produced dose-related inhibition on the mechanical allodynia. The number of animals corresponding to each of the testing points in each group is indicated in parentheses. (B, C) α-LA produced dose-related effects on MMP-9 and MMP-2 activity in the DRG (B) and DH (C). α-LA inhibited the increased MMP-9 and rescued the decreased MMP-2. Four samples from independent mice were included in each of the groups. (D) The spinal administration of MMP-2 produced a dose-related inhibition of mechanical allodynia induced by STZ treatment. (E) MMP-9^-/-^ mice exhibited mild mechanical allodynia following STZ treatment (see Fig. 4B) that was much less than in WT animals (see A). α-LA did not produce significant inhibition on the mechanical allodynia in MMP-9^-/-^ mice. (A-E) Twelve mice were included in each of the groups. (A-E) Data are expressed as the mean ± SEM. Two-way (A and E) and one-way ANOVA (B-D). A student’s t-test was used to test each of the specific points after the drug injection compared to the corresponding point immediately before the injection (A). **P < 0.01 versus vehicle (A-E); ##P < 0.01 versus the corresponding point immediately before the drug injection (A); ##P < 0.01 versus STZ treatment alone (B-D).

### Targeted mutation of MMP-9 alleviates STZ-induced myelin abnormalities and reverses myelin and mitochondria abnormalities

Axonal demyelination of the nerves can be caused by diabetes mellitus, nerve injury, or cobra venom, resulting in severe pain [39-42]. Unlike humans, diabetic rodents do not display consistent evidence of segmental demyelination in the peripheral nervous system, often leading to the impression that the lesion consists primarily of an axonal disturbance. We examined the possible morphological changes in the peripheral sciatic nerves and DH in WT and MMP-9-/- mice with and without STZ-induced DNP using an electron microscope. Electron micrographs showed that axons of the sciatic nerve from WT mice exhibited a fine, lightly stained filament network in the axoplasm. In contrast, the axons of the sciatic nerve from mice with DNP exhibited signs of severe pathological axonal abnormalities, such as reductions in the axon diameter and myelin thickness (Fig. 4A, left up). These changes in axonal dysfunction and myelin abnormalities were also observed in the DH (Fig. 4A, left down). These alterations in axonal abnormalities were significantly improved in MMP-9^-/-^ mice (Fig. 4A, right). Our detailed analysis of the changes in axonal degeneration and myelin and mitochondria abnormalities in the peripheral nerves and DH are shown in Fig. 4B-E. The decreased g-ratio (axonal diameter divided by the fiber diameter; Fig. 4B), decreased proportion of large, myelinated fibers (defined as fibers with a diameter > 6 μm; Fig. 4C), increased myelin abnormalities (Fig. 4D) in both peripheral nerves and the DH, and signs of mitochondrial swelling in the DH (Fig. 4E) in WT mice produced by STZ treatment were significantly reversed in MMP-9^-/-^ animals. We noticed no changes in the quantity and average area of mitochondria in the peripheral nerves and the number of mitochondria in the DH of WT or MMP-9^-/-^ mice following STZ treatment (Fig. 4E). Myelin basic protein marker (MBP) and myelin degraded markers (DMBP) were also used to confirm the integrity of myelin in wild-type and MMP-9^-/-^ mice. Our results showed that MBP expression was significantly decreased, and DMBP expression was significantly increased after STZ treatment, indicating that myelin degradation was increased. These alterations were significantly improved in the MMP-9^-/-^ mice (Fig. 4F). These findings demonstrate that targeted mutation of MMP-9 can prevent or reverse STZ-induced axonal dysfunction and protect myelin homeostasis of the peripheral fibers and DH, in addition to inhibiting DNP.

### Systematic administration of α-LA suppresses STZ-induced mechanical allodynia and MMP-9 activity in the DRG and DH

α-LA is a natural antioxidant synthesized in the mitochondria that is derived from food. α-LA supplementation can alleviate diabetic complications in humans and animals [43-45]. We wondered whether α-LA could reduce DNP and, if so, what its underlying mechanism could be. The results showed that the systematic administration of a single dose of α-LA (i.p., each at 30 mg/kg, 100 mg/kg, and 200 mg/kg, respectively) 21 days after STZ treatment produced a dose-dependent inhibition of the established STZ-induced mechanical allodynia in WT mice. The inhibition started within 2 h and lasted for approximately 4-6 h (30 mg/kg and 100 mg/kg) or 6-8 h (100 mg/kg) (Fig. 5A). Our gelatin zymography analysis showed that the activity of MMP-9 in the DRG (Fig. 5B) and DH (Fig. 5C) was significantly inhibited by the repetitive, systematic administration of α-LA in three groups treated with different doses of α-LA (10 mg/kg, 30 mg/kg, and 100 mg/kg, respectively, daily on days 19, 20, and 21). Interestingly, our results also showed that the decreased activity of MMP-2 in the DRG and DH induced by STZ could be significantly reversed by α-LA treatment (Fig. 5B, C, also see Fig. 2A, B). Thus, we wondered whether DNP could be alleviated by the spinal administration of MMP-2 since MMP-2i failed to inhibit DNP (see Fig. 1E, F). Our results showed that a single dose of MMP-2 (i.t., 0.2, 0.4, and 0.8 pmol, respectively) administrated 21 days after STZ treatment produced a dose-related inhibition of the established STZ-induced mechanical allodynia in WT mice with DNP (Fig. 5D). However, as expected, a single dose of MMP-2 (i.t., 0.2 pmol) in naïve WT mice without STZ or any other treatment produced mechanical allodynia (algesic, not analgesia; Fig. 5D), which is consistent with a previous report [17]. We further tested the pharmacological effects of α-LA on STZ-induced DNP in MMP-9^-/-^ mice, which showed significantly decreased mechanical allodynia following STZ treatment compared with WT animals (see Fig. 3C). The results showed that a single dose of α-LA at the higher dose of 100 mg/kg (i.p.), which produced significant inhibition of mechanical allodynia in WT mice with DNP (see Fig. 5A), did not significantly produce further inhibition of mechanical allodynia in MMP-9^-/-^ mice. However, some inhibition was observed (Fig. 5E). These results suggest that the systematic administration of α-LA can suppress DNP likely by inhibiting the increased MMP-9 and rescuing the decreased MMP-2 in the DRG and DH.

## DISCUSSION

**Figure 6.**
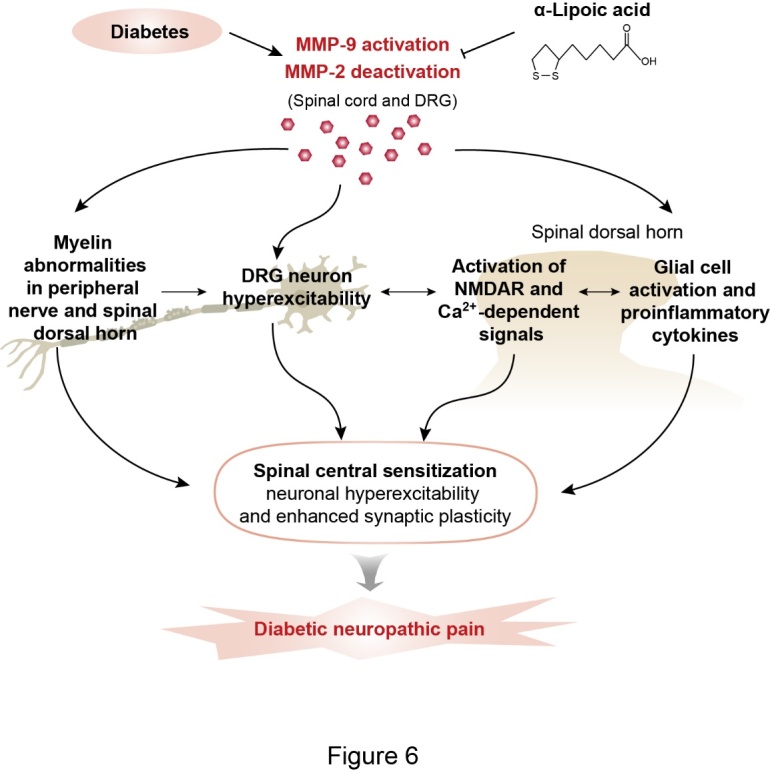
Schematic representation of the mechanisms of gelatinase MMP-9 and MMP-2 in the DRG and spinal cord underlying diabetic neuropathic pain (DNP) and α-LA inhibition of DNP. The flowchart illustrates the possible pathways for MMP-9 signaling-induced spinal central sensitization and DNP and the possible functional link between MMP-9 activation, MMP-9-dependent cellular responses, interactions between MMP-9 and NMDAR and its subsequent signaling pathways, glial cell activation, the activity of proinflammatory cytokines, myelin abnormalities, and behavioral manifestations of diabetic neuropathic pain. MMP-2 contributes to DNP and the α-LA-induced inhibition of DNP by serving as a negative regulator. α-LA inhibits DNP by inhibiting MMP-9 activation and rescues MMP-2 deactivation.

Our study reveals a critical role for gelatinase MMP-9 and MMP-2 in myelin abnormalities and neuropathic pain in rodents with STZ-induced diabetic neuropathy. After STZ treatment, the activity of MMP-9 increased, while that of MMP-2 decreased in the DRG and DH. MMP-9 may contribute to DNP by inducing myelin abnormalities of peripheral fibers and DH and increasing the phosphorylation of NMDAR and the subsequent activation of Ca^2+^-dependent downstream signaling pathways. MMP-2 may contribute to DNP by serving as a negative regulator. The major findings are 4-fold. First, in STZ-DNP animals, the activity of MMP-9 was increased in the nociceptive DRG neurons as well as neurons and astrocytes in the DH. Meanwhile, gelatinase MMP-2 activity was significantly decreased in the DRG and DH. Second, spinal inhibition of MMP-9, but not MMP-2, suppressed the behavioral and neurochemical signs of DNP. In contrast, spinal administration of MMP-2 alleviated DNP. Third, axonal degeneration and myelin abnormalities were seen in the peripheral sciatic nerves and DH, along with mitochondrial swelling in the DH of mice with DNP. These neuropathic alterations were much improved in MMP-9^-/-^ mice, which still exhibited milder DNP. Fourth, the systematic administration of a natural antioxidant α-LA suppressed DNP, inhibited MMP-9 activity, and rescued the activity of MMP-2 in the DRG and DH. These findings support a new mechanism underlying the pathogenesis of diabetic neuropathy and suggest a potential target for DNP treatment. This study also suggests that the natural antioxidant α-Lipoic acid may be a potent analgesic for DNP. The model of MMP-9 and MMP-2 signaling in the DRG and the spinal cord underlying the DNP and the α-LA-induced inhibition of the DNP are illustrated in Fig. 6.

MMP-9 and MMP-2 are expressed in the brain, spinal cord, and DRG, and are often increased in response to inflammation, injury, or neurological diseases [17, 21, 46, 47]. Analysis of the cellular distribution of MMP-9 and MMP-2 has revealed that MMP-9, but not MMP-2, increases in neurons and astrocytes in the rat hippocampus following intraperitoneal kainate administration [48], as well as in DRG neurons and astrocytes after spinal nerve ligation [17] or morphine withdrawal [21], while MMP-2 activity increases in astrocytes in the DH [17]. The present study demonstrated that MMP-9 activity was significantly increased, while MMP-2 activity was greatly decreased in both the DRG and DH in DNP animals. Increased MMP-9 activity occurs mainly in the superficial layers of the DH, an essential area for the generation and processing of pain signals, colocalizing primarily with neurons and astrocytes. In the DRG, increased MMP-9 activity colocalizes with the CGRP- and IB4-positive nociceptive neurons. This distribution of MMP-9 is similar to that reported in the spinal nerve ligation (SNL) model [17], but different from that in the model of pain enhancement after morphine withdrawal, where MMP-9 activity was increased in the DH but not in the DRG, as we have reported [21]. In contrast, the activity of MMP-2 was significantly and consistently decreased in the DRG and DH in DNP animals in the current study. This is consistent with a previous report that MMP-2 activity was decreased in the sciatic nerve after STZ treatment [22]. However, MMP-2 activity was found to be greatly increased in the DRG and spinal cord after SNL [17], with no changes found in the DRG and DH following morphine treatment and withdrawal [21]. Overall, MMP-9 and MMP-2 activity may respond and contribute differently in the context of different pathological conditions. In addition to the different changes and distributions of MMP-9/2 in different models, our results also show that, in STZ-treated animals, MMP-9 activation in the DRG and spinal cord was increased two to three weeks after the STZ injection but was not altered during the early period of DNP production. Thus, the activity of MMP-9 in the spinal cord may be involved in established DNP, but not in the early period of DNP production. Corresponding to such timing alterations, the spinal inhibition of MMP-9 significantly inhibits established ongoing mechanical allodynia but fails to prevent the induction of DNP.

NMDARs play a well-established role in neural plasticity and various pain states. NMDAR activation results in Ca^2-^ influx through the NMDAR ion channel complex [36, 37]. Subsequent activation of various Ca^2-^-dependent signaling enzymes, such as CaMKII, can phosphorylate CREB, leading to increased levels of proinflammatory cytokines IL-1β and TNF-α [32, 36-38]. We have provided evidence that STZ treatment increases the phosphorylation of NMDAR NR1 subunit and CaMKII, PKC, ERK, and CREB. STZ treatment also induces the activation of astrocytes and microglial cells and the expression of the proinflammatory cytokines IL-1β and TNF-α. Spinal blocking of MMP-9 activity or targeted mutation of MMP-9 significantly suppresses the neurochemical signs and behaviorally expressed mechanical allodynia in DNP rats and mice, respectively. Spinal administration of MMP-2, but not MMP-2 inhibitor, can reduce mechanical allodynia, suggesting that MMP-2 deficits may contribute to DNP and that providing exogenous MMP-2 to replenish the deficits can help alleviate DNP. Our results further support that the systematic administration of α-LA reduces mechanical allodynia and rescues MMP-2 activity in the DH. These findings support the idea that MMP-9 and MMP-2, serving as positive and negative regulators, respectively, are involved in the maintenance of STZ-induced DNP.

In diabetes, sustained hyperglycemia can result in neuropathy, including myelin abnormalities and degeneration in peripheral fibers that can directly lead to central spinal sensitization and DNP [39, 49-52]. Many patients with diabetes experience spontaneous pain and evoke mechanical allodynia. Sometimes, the pain is severe and accompanied by other symptoms related to sensory and autonomic disturbances [53, 54]. Here, we have proven that axons of sciatic nerves and the DH in DNP mice exhibit clear signs of axonal degeneration as well as myelin and mitochondria abnormalities. Interestingly, targeted mutation of MMP-9 can improve or reverse these changes and alleviate DNP severity. These findings support the idea that increased activity of MMP-9 may be involved in mediating the development of myelin and axonal abnormalities that accompany sustained hyperglycemia and thus contribute to DNP.

Another interesting finding in the current study is that the systematic administration of α-LA can suppress mechanical allodynia, inhibit MMP-9 activity, and rescue MMP-2 activity in the DRG and DH of DNP animals. These findings support the use of α-LA in DNP treatment and demonstrate that α-LA alleviates DNP at least partly by inhibiting STZ treatment-induced MMP-9 activity and rescuing MMP-2 deficits in the DRG and DH. α-LA is a natural antioxidant that is synthesized in the mitochondria and can be derived from food. Interestingly, previous studies have suggested that α-LA supplementation can alleviate diabetic complications in humans and animals [43-45]. In addition, this study also showed that α-LA could attenuate mechanical allodynia in WT mice but not produce further inhibition of mechanical allodynia in MMP-9^-/-^ mice, which already showed significantly milder DNP (approximately 80% less intense than WT mice). These findings suggest that MMP-9 may serve as the main mechanism underlying the α-LA-induced inhibition of DNP.

Our study indicates that the gelatinases MMP-9 and MMP-2 play a critical role in DNP and the associated myelin abnormalities in rodents. MMP-9 may contribute to STZ treatment-induced DNP by its involvement in myelin abnormalities and central spinal sensitization, while MMP-2 may serve as a negative regulator. These findings support a new mechanism underlying the pathogenesis of diabetic neuropathy and suggest a potential target for DNP treatment. Given the importance of MMP-9 and MMP-2 in many neuroinflammation-related diseases and the excellent clinical safety history and low cost of α-LA, our findings may represent a bright prospect for the development of MMP-9 inhibitors based on α-LA. Furthermore, our findings also provide a new perspective and basis for the development of novel analgesic and anti-inflammatory drugs using α-LA as a lead compound.
